# Microbiota in Human Periodontal Abscess Revealed by 16S rDNA Sequencing

**DOI:** 10.3389/fmicb.2019.01723

**Published:** 2019-07-30

**Authors:** Jiazhen Chen, Xingwen Wu, Danting Zhu, Meng Xu, Youcheng Yu, Liying Yu, Wenhong Zhang

**Affiliations:** ^1^Department of Infectious Diseases, Huashan Hospital, Fudan University, Shanghai, China; ^2^Department of Dentistry, Zhongshan Hospital, Fudan University, Shanghai, China; ^3^Department of Dentistry, Huashan Hospital, Fudan University, Shanghai, China

**Keywords:** high-throughput sequencing, oral microbiota, periodontal abscess, 16S rDNA metagenomic, periodontal pocket

## Abstract

Periodontal abscess is an oral infective disease caused by various kinds of bacteria. We aimed to characterize the microbiota composition of periodontal abscesses by metagenomic methods and compare it to that of the corresponding pocket and healthy gingival crevice to investigate the specific bacteria associated with this disease. Samples from abscess pus (AB), periodontal pocket coronally above the abscess (PO), and the gingival crevice of the periodontal healthy tooth were obtained from 20 periodontal abscess patients. Furthermore, healthy gingival crevice samples were obtained from 25 healthy individuals. Bacterial DNA was extracted and 16S rRNA gene fragments were sequenced to characterize the microbiota and determine taxonomic classification. The beta-diversity analysis results showed that the AB and PO groups had similar compositions. *Porphyromonas gingivalis*, *Prevotella intermedia*, and other *Prevotella* spp. were the predominant bacteria of human periodontal abscesses. The abundances of *Filifactor alocis* and *Atopobium rimae* were significantly higher in periodontal abscesses than in the periodontal pocket, suggesting their association with periodontal abscess formation. In conclusion, we characterized the microbiota in periodontal abscess and identified some species that are positively associated with this disease. This provides a better understanding of the components of periodontal abscesses, which will help facilitate the development of antibiotic therapy strategies.

## Introduction

Periodontal abscess is an acute exacerbation of chronic periodontitis, exhibiting clinical symptoms of swelling and severe pain in the gingival margin. It is defined as a localized suppurative lesion that is related to periodontal alveolar bone loss and the accumulation of pus in the gingival wall of the periodontal pocket (Herrera et al., 2000). Previously, we cultured obligate anaerobic bacteria from periodontal abscess and characterized their antimicrobial resistance profiles (Xie et al., 2014), in which the predominant obligate anaerobes were black-pigmented *Prevotella*. Although the results were partly in agreement with the findings of previous studies (Jaramillo et al., 2005; Herrera et al., 2014), some bacteria such as those of the genus *Treponema* were unculturable and some predominant anaerobes such as *Porphyromonas gingivalis*, *Tannerella forsythia*, and *Fusobacterium* spp. were less frequently cultured, due to the culture condition or suitability of medium (Xie et al., 2014). In addition, it has been reported that the therapeutic effect of antibiotic regimens on periodontal abscess is limited (Smith and Davies, 1986; Herrera et al., 2000, 2014), suggesting the complexity of associated pathogens. Recently, we used high-throughput barcoded 16S rDNA sequencing to characterize the microbiota in the periodontal pocket of patients with periodontitis and compared these to those of patients with chronic obstructive pulmonary disease (COPD). As the number of different kinds of bacteria was determined in the subgingival plaque of every patient, we hypothesized that periodontal abscess is caused by a combination of microbiota, and specific pathogens might be more dominant in the abscess than in the pocket and in healthy controls suggesting a positive-association with abscess formation. Therefore, a clearer understanding of pathogens and the microbiota that cause periodontal abscess is necessary.

In this study, we used high-throughput barcoded 16S rDNA sequencing technique to characterize the microbiota of periodontal abscess, the corresponding pocket, and healthy gingival crevice to investigate the specific bacteria associated with periodontal abscess in human periodontitis.

## Materials and Methods

### Patient Recruitment

Forty-five participants were recruited from March 2015 to September 2015, including 20 periodontitis patients with periodontal abscess and 25 periodontal healthy individuals. Subjects with the following conditions were excluded from the study: pregnancy, use of antibiotics or anti-inflammatory drugs during the past 3 months, and administration of periodontal therapy during the last 6 months. The study was approved by the ethics committee of Huashan Hospital, Fudan University (No. KY2014-023). All participants provided signed, informed consent. The study design is shown in Supplementary Figure S1. Probing depth (PD), clinical attachment loss (CAL), and simplified oral hygiene index (OHI-S) were assessed according to World Health Organization recommendations (WHO, 1997). Periodontitis was diagnosed as previously described (Wu et al., 2017) with the presence of more than one tooth with at least one site (mesiobuccal, buccal, distobuccal, mesiolingual, lingual, and distolingual sites) with PD ≥ 4 mm, CAL ≥ 2 mm, and bleeding on probing. Periodontal abscess was diagnosed by a periodontal specialist based on the patients’ symptomatology, clinical and radiological examination findings such as swelling and enlargement of the gingiva, history of periodontal disease, and radiograph of the alveolar bone destruction around the cementoenamel junction. Patients with periodontal abscess but without periodontitis were excluded. Periodontal tooth health was defined as PD ≤ 2 mm and with no bleeding on probing at all six sites.

### Specimen Collection and Isolation of Bacterial DNA

Three samples were collected from all the patients with abscess, including the sample of abscess pus (abscess group, AB), periodontal pocket coronally above the abscess (pocket group, PO), and gingival crevice of periodontal healthy tooth (patient control group, PC). Healthy teeth were also sampled from periodontally healthy individuals as the healthy control (control group, HC). The abscess samples were drained after decontamination of the mucosa. A No. 25# sterilized paper point (Gapadent, China) was immersed into the deep area of pus for 10 s after drainage with a sterilized probe. The periodontal pocket and healthy gingival crevice samples were dipped with No. 25# sterilized paper points as previously described (Wu et al., 2017). The periodontal pocket sample was not collected from one patient due to contamination with pus. All samples were stored in tris-EDTA buffer solution of pH 7.4 (Sigma, United States) in a freezer (−80°C). Bacterial DNA was extracted using the QiAamp DNA Mini Kit (Qiagen, Germany), according to the manufacturer’s instructions.

### Amplification of the 16S rDNA and Sequencing

According to previous studies (Claesson et al., 2010; Mizrahi-Man et al., 2013; Jorth et al., 2014), hypervariable V3–V4 or V4–V5 regions are recommended to study the microbiome when using the second-generation sequencing method. It was also determined that the V4–V5 region showed the best performance among all regions. Therefore, the amplification of the V4–V5 regions of 16S rDNA, library construction, index PCR, and PCR clean-up were performed as previously described (Wu et al., 2017). Equal amounts of tagged 16S rRNA gene amplicons of each sample were mixed and denatured with 0.1 M NaOH. The mixed library was diluted to a final concentration of 10–20 pM using 10 mM tris at pH 8.5. Multiplexed paired-end sequencing (2 × 300 bp reads) of the 16S rRNA amplicons was performed using a Miseq system (Illumina, San Diego, CA, United States). Image analysis and base calling were performed on the Miseq system using the MiSeq Reporter software (MSR). After de-multiplexing the data and removing the reads that failed the purity filter (PF = 0), the reads were converted to FASTQ format.

### Data Analyses

The generated FASTQ files (.fastq) and quality files were acquired as raw and mapped sequence data using default settings of the QIIME2 software (version: 2018.8) (Caporaso et al., 2010). Each operational taxonomic unit (OTU) was generated with 97% similarity cutoff using UPARSE v7.1 and chimeric sequences were identified and removed using UCHIME. The phylogenetic affiliation of each 16S rRNA gene sequence was analyzed using RDP Classifier^1^ based on the Silva (SSU132) 16S rRNA database using a confidence threshold of 70% (Amato et al., 2013; Duan X.B. et al., 2017; Duan X. et al., 2017; Xu et al., 2018). The output was based on the classification of reads at several taxonomic levels. The alpha- and beta-diversity analyses were computed from the previously constructed OTU table using Mothur software (v.1. 30.1) (Schloss et al., 2009) and weighted UniFrac (Lozupone et al., 2011) analysis. In the group level, abundance analysis was determined from rarefaction files by the Mann–Whitney test between patient and health control groups and a paired *t*-test in the self-control comparison (SPSS Statistics v20.0 and GraphPad Prism software v6.01). At the patient level, when analyzing the significantly dominant bacteria in abscess patients, any OTUs with abundance differences greater than 10% were considered significantly dominant. The minimum abundance cutoff was set at 0.1% abundance, and abundance values < 0.1% were neglected.

## Results

### Participant Characteristics

Characteristics of the 45 enrolled subjects are listed in Table 1. The sex proportion and smoking status between patients and controls were not statistically different.

**TABLE 1:** Characteristics data of the enrolled study participants.

	**Patient (*N* = 20)**	**Health control (*N* = 25)**
**Gender (%)**		
Male	11 (55.0%)	11 (44.0%)
Female	9 (45.0%)	14 (56.0%)
**Age**		
Mean (sd)	53.2 (16.2)	64.8 (6.7)
Smoking status (%)		
Non-smoker^a^	17 (85.0%)	20 (80.0%)
Smoker	3 (15.0%)	5 (20.0%)
Former smoker^b^	0	1 (4.0%)
Current smoker^c^	3 (15.0%)	4 (16.0%)
**Cigarettes/day, Mean (SD)**		
Smoker	12.3 (7.5)	16.4 (14.4)
Former smoker	0	20 (N/A)
Current smoker	12.3 (7.5)	15.5 (16.5)
**Periodontal Index**		
PD, Mean (SD)	4.8 (0.7)	2.5 (0.5)
CAL, Mean (SD)	5.8 (1.0)	2.8 (0.7)
OHI-S, Mean (SD)	2.43 (1.12)	1.45 (0.51)

*^*a*^Non-smokers were those who either had never smoked or quit cigarettes at least 10 years prior to study entry. ^*b*^Former smokers were those who quit cigarettes at least 6 months but < 10 years prior to study entry. ^*c*^Current smokers were currently smokers or those who quit cigarettes < 6 months prior to study entry. PD, probing depth; CAL, clinical attachment loss; OHI-S, simplified oral hygiene index.*

### Taxonomic Classification of the 16S rDNA Sequences

In total, 4.1 GB raw data containing 1.70 million high-quality and classifiable reads were obtained from 84 samples. The sequencing depth was similar among the four groups as follows: 20.85 ± 3.16 k reads in the AB group, 15.50 ± 1.93 k reads in the PO group, 15.40 ± 1.40 k reads in the PC group, and 27.31 ± 7.21 k reads in the HC group. Among these high-quality reads, 99.41% were classified into 322 genera, belonging to 22 phyla, 38 classes, 84 orders, and 153 families. There was no significant difference in the proportion of unclassifiable sequences among the four groups based on a Kruskal–Wallis test (*P* = 0.186).

### Alpha-Diversity Analysis

The alpha-diversity analysis was conducted with two indexes, namely the Shannon index implicating community diversity and the Chao1 index implicating community richness (Supplementary Figure S2). Plots were generated and exported to the rarefaction curves (Aagaard et al., 2012; Supplementary Figure S3). There was no significant difference in Chao1 and Shannon indexes among the four groups based on a one-way ANOVA analysis with Tukey’s multiple comparisons test.

### Beta-Diversity Analysis

**FIGURE 1:**
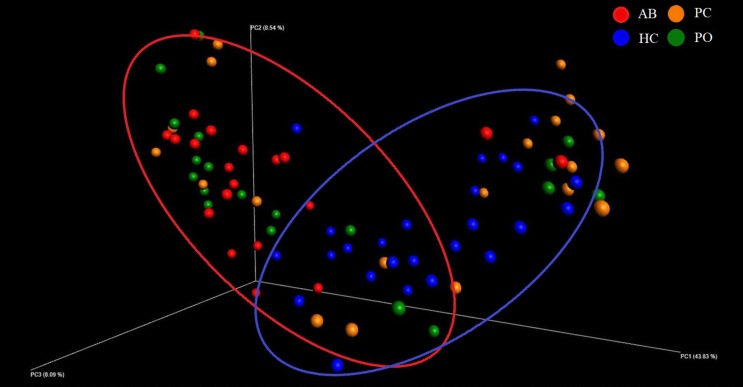
Beta diversity analysis based on UniFrac analysis. Plots were generated using weighted UniFrac distances. Red dot represents the abscess pus (AB) group. Green dot represents the pocket (PO) group. Yellow dot represents the patient control (PC) group. Blue dot represents the healthy control (HC) group. Circles in red and blue represent different periodontal bacterial community clusters, respectively.

The microbial raw OTU data were subjected to the principal coordinate analysis (PCoA) to evaluate the similarities among the four groups (Figure 1). The results showed that the samples from the AB and PO groups had similar microbiota compositions, which could be grouped into one cluster, whereas the HC group formed another cluster. The samples from the PC group could not be grouped into one cluster and were scattered in the 3D plot (Figure 1).

### Abundance Analysis

**FIGURE 2:**
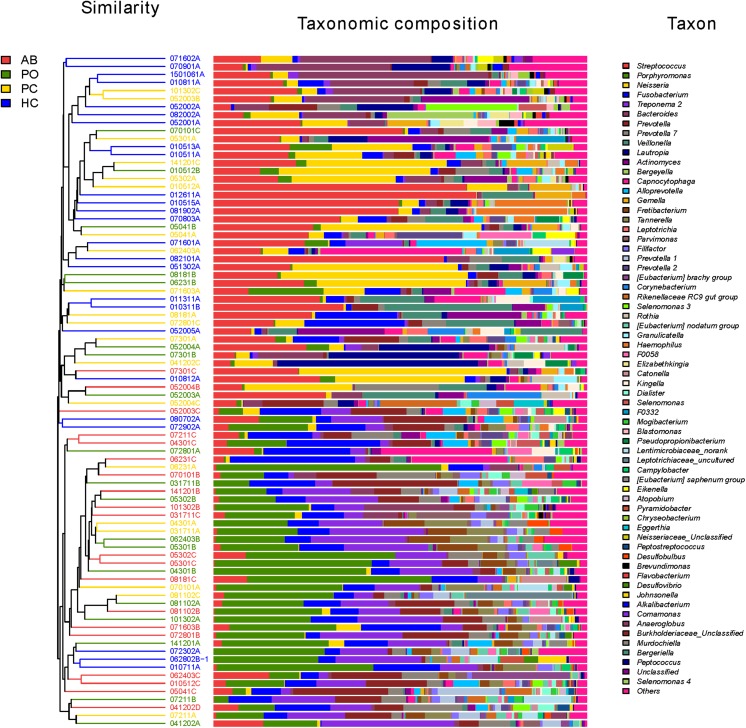
Microbial community bar plot at the genus level with cluster tree. Each line shows the results of the bacterial community of one sample. Different bacterial taxonomies at the genus level (cutoff was set as 1%; some below 1% were shown as “others”) are shown in different colors on the right. The hierarchical cluster diagrams in all samples based on the community composition (based on the algorithm of Bray–Curtis) is shown on the left. Red color indicates that the specimen is in the AB group. Green color indicates that the specimen is in the pocket (PO) group. Yellow color indicates that the specimen is in the healthy control (HC). Blue color indicates that the specimen is in the patient control (PC).

Consistent with the results of the beta-diversity analysis, 8 of the top 10 most dominant bacteria were the same in these two groups. The genera *Porphyromonas*, *Treponema 2*, *Streptococcus*, *Neisseria*, *Fusobacterium*, *Prevotella*, *Prevotella 7* and *Tannerella* accounted for 61% of the bacteria in the AB and PO groups, and exhibited no significant differences between the AB and PO groups based on a paired *t*-test (Table 3 and Supplementary Figure S4). Furthermore, based on the hierarchical clustering analysis of the four groups, 17 of the 20 (85%) AB samples and 11 of the 19 (58%) PO samples clustered together (lower cluster in Figure 2). In the PC and HC control groups, 8 of the top 10 most dominant bacteria overlapped. The genera *Streptococcus*, *Neisseria*, *Bacteroides*, *Fusobacterium*, *Veillonella*, *Prevotella*, *Actinomyces*, and *Porphyromonas* accounted for 57 and 59% of the total bacteria in the PC and HC groups, respectively (Supplementary Figure S4). Fourteen of the 20 (70%) PC samples and 22 of the 25 (88%) HC samples clustered together (upper cluster in Figure 2), indicating almost similar compositions between these two groups.

### Dominant Bacteria in Abscesses at the Patient Level

**FIGURE 3:**
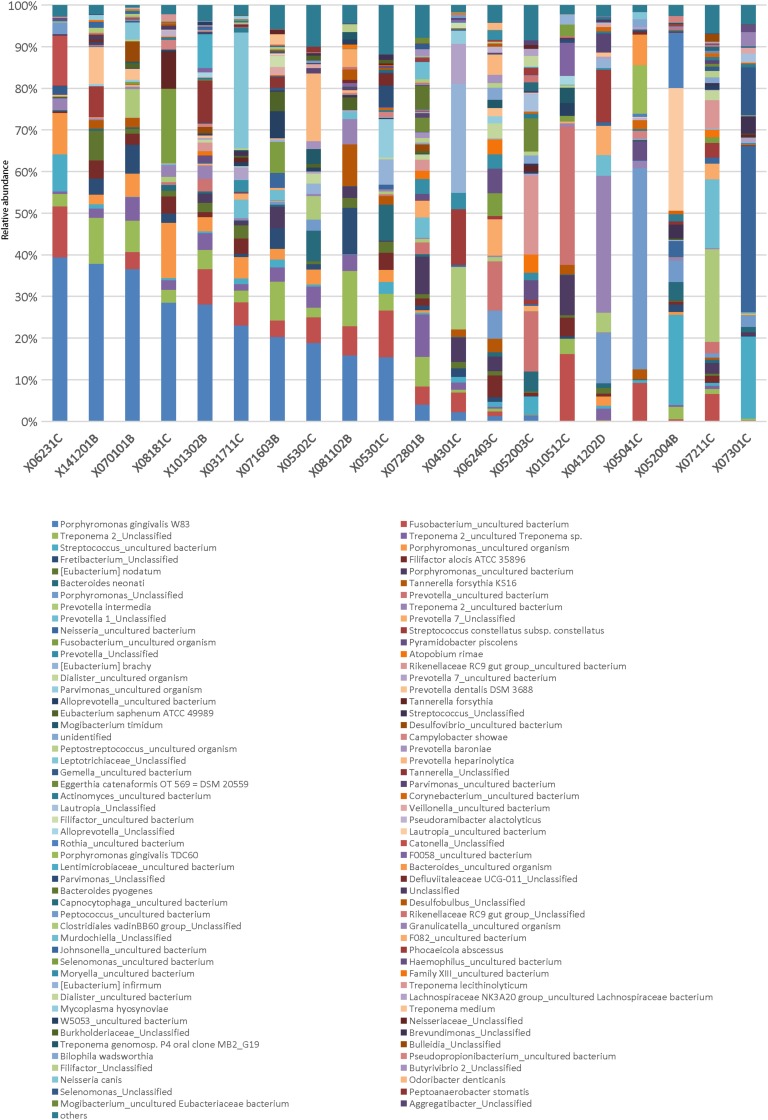
Relative abundance of genera in the AB group. Each column represents a sample in the AB group. The genera were sorted in descending order of average abundance in abscess samples. Different bacterial taxonomies at the genus level are shown in different colors on the bottom. Abundance cutoff in this bar graph was set at 1%; some below 1% were shown as “others.”

In the AB group, the abundance of the most abundant OTUs in all samples did not exceed 50% (Figure 3). The data showed that 9 of 20 samples had two OTUs with an abundance over 10% and 7 of 20 samples had three or more OTUs with an abundance over 10%. Furthermore, 2 of 20 samples had two OTUs with an abundance over 20%, including the combination of *Leptotrichiaceae_Unclassified* (27.8%) and *P. gingivalis W83* (23.1%) in abscess 031711C, and *Lautropia_uncultured bacterium* (29.5%) and *Streptococcus_uncultured bacterium* (21.6%) in abscess 052004B (Figure 3). These results suggest that disease in these patients was caused by bacterial co-infections.

To reveal the dominant bacteria involved in abscess, we compared the OTUs of the AB group with those of the PC group by performing a paired *t*-test at the group level. The results showed that the abundance of 6 OTUs was significantly higher in the AB than in the PC group (Supplementary Table S1). However, except *P. gingivalis W83*, other classical opportunistic bacteria causing periodontal abscess were not significantly different based on this analysis. Furthermore, the OTUs, except *P. gingivalis W83*, which was relatively high in the AB group, were low in average abundance (Supplementary Table S1), suggesting that group comparison is not ideal for the analysis of dominant bacteria.

Considering the heterogeneity of dominant opportunistic bacteria in different patients, we performed a direct bacterial abundance comparison between the AB and PC groups at the patient level, and the bacteria with abundance differences > 10% between the AB and PC groups were identified as significantly dominant bacteria (Table 2 and Supplementary Table S2). In total, 19 OTUs including *P. gingivalis W83* (8/20, 40%), *Prevotella* spp. (3/20, 15%), *Prevotella intermedia* (2/20, 10%), *P. gingivalis TDC60* (1/20, 5%), and *Prevotella heparinolytica* (1/20, 5%), were found to be significantly dominant in the AB group compared with abundances in the PC group in the corresponding number of patients (Table 2). Additionally, 20 OTUs, including *Streptococcus* spp. (6/20, 30%)*, Actinomyces* spp. (3/20, 15%)*, Lautropia* spp. (3/20, 15%), *Neisseria* spp. (3/20, 15%), *Veillonella* spp. (3/20, 15%), *Fusobacterium* spp. (2/20, 10%), *P. intermedia* (2/20, 10%), and *Bacteroides neonati* (2/20, 10%), were found to be significantly more dominant in the PC group than in AB group (Table 2). Interestingly, *P. intermedia* was identified as significantly dominant in the AB group of some patients and the PC group of other patients, indicating that it might have a heterogeneous function in abscess formation in different populations.

**TABLE 2:** The Dominant OTUs in AB group compared to PC group at patient level.

**OTUs**	**Patients (Radio)**
**Greater than 10% higher in AB group than in PC group**	
*Porphyromonas gingivalis W83*	8 (40%)
*Prevotella_uncultured bacterium*	3 (15%)
*Prevotella intermedia*	2 (10%)
*Streptococcus constellatus subsp. constellatus*	2 (10%)
*Fusobacterium_uncultured bacterium*	2 (10%)
*Porphyromonas_Unclassified*	2 (10%)
*Porphyromonas gingivalis TDC60*	1 (5.0%)
*Prevotella heparinolytica*	1 (5.0%)
*[Eubacterium] brachy*	1 (5.0%)
*Fusobacterium_uncultured organism*	1 (5.0%)
*Leptotrichiaceae_Unclassified*	1 (5.0%)
*Porphyromonas_uncultured organism*	1 (5.0%)
*Prevotella 1_Unclassified*	1 (5.0%)
*Rikenellaceae RC9 gut group_uncultured bacterium*	1 (5.0%)
*Streptococcus_uncultured bacterium*	1 (5.0%)
*Tannerella_Unclassified*	1 (5.0%)
*Treponema 2_Unclassified*	1 (5.0%)
*Treponema 2_uncultured Treponema* sp.	1 (5.0%)
*Treponema 2_uncultured bacterium*	1 (5.0%)
**Greater than 10% lower in AB group than in PC group**	
*Streptococcus_uncultured bacterium*	6 (30%)
*Actinomyces_uncultured bacterium*	3 (15%)
*Lautropia_uncultured bacterium*	3 (15%)
*Neisseria_Unclassified*	3 (15%)
*Veillonella_uncultured bacterium*	3 (15%)
*Prevotella intermedia*	2 (10%)
*Bacteroides neonati*	2 (10%)
*Fusobacterium_uncultured bacterium*	2 (10%)
*Porphyromonas gingivalis W83*	1 (5.0%)
*Bergeriella denitrificans*	1 (5.0%)
*Treponema 2_Unclassified*	1 (5.0%)
*Capnocytophaga_uncultured bacterium*	1 (5.0%)
*Corynebacterium_uncultured bacterium*	1 (5.0%)
*Fretibacterium_Unclassified*	1 (5.0%)
*Gemella_uncultured bacterium*	1 (5.0%)
*Haemophilus_uncultured bacterium*	1 (5.0%)
*Lentimicrobiaceae_uncultured bacterium*	1 (5.0%)
*Neisseria_uncultured bacterium*	1 (5.0%)
*Porphyromonas_uncultured bacterium*	1 (5.0%)
*Streptococcus_Unclassified*	1 (5.0%)

*The dominant different abundance of OTU was set as 10%.*

### Specific Bacteria in Abscess Compared With Those in Pockets at the Group Level

At the group level, a comparison between the AB and PO groups revealed specific bacteria associated with acute disease. The abundance of bacteria in these two groups was highly similar. At the genus level, only *Filifactor* and *Atopobium* exhibited significantly higher abundance in the AB group, whereas nine genera presented significantly lower in abundance in the AB group than in the PO group (Table 3). Similarly, 3 OTUs including *Filifactor alocis* and *Atopobium rimae* presented significantly higher abundance in the AB group, suggesting that they might function in periodontal abscess formation. Moreover, 4 OTUs exhibited significantly lower abundance in the AB group than in the PO group (Table 3). However, all 4 OTUs were not accurately classified to the species level.

**TABLE 3:** Specific bacteria of significant difference in AB compared with PO group based on a paired *t*-test at group level.

**Genus/OTUs**	**AB (Mean ± SE, %)**	**PO (Mean ± SE, %)**	***p*-value**
**Higher in AB in Genus level**			
*Filifactor*	2.05 ± 0.40	1.06 ± 0.26	0.009
*Atopobium*	0.75 ± 0.33	0.03 ± 0.01	0.046
**Lower in AB in Genus level**			
*Leptotrichia*	0.15 ± 0.05	0.96 ± 0.25	0.005
*Selenomonas 3*	0.12 ± 0.06	0.75 ± 0.29	0.029
*Desulfobulbus*	0.24 ± 0.13	0.61 ± 0.22	0.019
*Prevotella 2*	0.06 ± 0.02	0.52 ± 0.14	0.009
*Flexilinea*	0.09 ± 0.04	0.44 ± 0.16	0.048
*Eikenella*	0.02 ± 0.01	0.23 ± 0.09	0.037
*F0332*	0.03 ± 0.02	0.12 ± 0.05	0.029
*Bergeyella*	0.04 ± 0.02	0.11 ± 0.04	0.012
*Lachnoanaerobaculum*	0.02 ± 0.01	0.10 ± 0.03	0.013
**Higher in AB in OTU level**			
*Streptococcus constellatus subsp. constellatus*	2.08 ± 0.97	0.36 ± 0.17	0.049
*Filifactor alocis ATCC 35896*	1.88 ± 0.43	0.77 ± 0.24	0.007
*Atopobium rimae*	0.68 ± 0.31	0.00 ± 0.00	0.039
**Lower in AB in OTU level**			
*Capnocytophaga_uncultured bacterium*	0.26 ± 0.11	1.82 ± 0.67	0.027
*Veillonella_uncultured bacterium*	0.20 ± 0.09	1.42 ± 0.61	0.040
*Desulfobulbus_Unclassified*	0.24 ± 0.13	0.61 ± 0.22	0.019
*Leptotrichia_uncultured bacterium*	0.12 ± 0.04	0.55 ± 0.19	0.034

*Abundance cutoff was set at 0.1%, some below 0.1% were not shown in the table.*

### Bacteria Associated With Abscess at the Group Level

At the group level, a comparison between the AB and HC groups was made in the present study. At the genus level, 24 genera including *Porphyromonas*, *Treponema 2*, *Tannerella*, *Filifactor*, *Parvimonas*, and *Prevotella 1* were significantly more abundant in the AB group than in the HC group (Supplementary Table S3). Moreover, 25 genera including *Streptococcus*, *Neisseria*, *Veillonella*, *Capnocytophaga*, *Actinomyces*, *Selenomonas 3*, and *Prevotella 2* were significantly less abundant in the AB group than in the HC group (Supplementary Table S3). At the OTU level, 28 OTUs including *P. gingivalis*, *Treponema 2* spp., *P. intermedia, F. alocis*, and *T. forsythia* exhibited significantly higher abundance in the AB group than in the HC group. In contrast, 22 OTUs including *Streptococcus* spp., *Veillonella* spp., *Actinomyces* spp., and *Neisseria* spp. showed less abundance in the AB group than in the HC group (Table 4).

**TABLE 4:** Mean relative abundance of OTUs with significant statistical difference between AB and HC groups at group level.

**OTUs**	**AB (Mean ± SE, %)**	**HC (Mean ± SE, %)**	***p*-value**
**Higher in AB**			
*Porphyromonas gingivalis W83*	13.64 ± 3.25	0.09 ± 0.04	< 0.001
*Treponema 2_Unclassified*	3.91 ± 0.86	0.75 ± 0.30	< 0.001
*Prevotella intermedia*	2.94 ± 1.31	0.17 ± 0.07	0.022
*Treponema 2_uncultured bacterium*	2.74 ± 1.62	0.46 ± 0.19	0.003
*Porphyromonas_uncultured organism*	2.63 ± 0.81	0.00 ± 0.00	< 0.001
*Treponema 2_uncultured Treponema* sp.	2.25 ± 0.58	0.08 ± 0.03	< 0.001
*Streptococcus constellatus subsp. constellatus*	1.98 ± 0.93	0.09 ± 0.05	0.006
*[Eubacterium] brachy*	1.95 ± 1.32	0.15 ± 0.07	0.018
*Fretibacterium_Unclassified*	1.90 ± 0.64	0.20 ± 0.09	0.004
*Filifactor alocis ATCC 35896*	1.79 ± 0.42	0.05 ± 0.03	< 0.001
*Prevotella 1_Unclassified*	1.77 ± 0.87	0.01 ± 0.01	0.003
*Rikenellaceae RC9 gut group_uncultured bacterium*	1.53 ± 0.99	0.00 ± 0.00	0.006
*[Eubacterium] nodatum*	1.37 ± 0.37	0.03 ± 0.02	< 0.001
*Tannerella forsythia KS16*	1.34 ± 0.52	0.05 ± 0.03	0.005
*Prevotella heparinolytica*	1.10 ± 0.83	0.00 ± 0.00	0.022
*Eubacterium saphenum ATCC 49989*	0.59 ± 0.27	0.02 ± 0.01	0.002
*Parvimonas_uncultured bacterium*	0.46 ± 0.23	0.02 ± 0.01	< 0.001
*Defluviitaleaceae UCG-011_Unclassified*	0.29 ± 0.14	0.03 ± 0.02	0.002
*Rikenellaceae RC9 gut group_Unclassified*	0.23 ± 0.07	0.00 ± 0.00	< 0.001
*Clostridiales vadinBB60 group_Unclassified*	0.22 ± 0.08	0.01 ± 0.01	0.003
*Pseudoramibacter alactolyticus*	0.19 ± 0.09	0.00 ± 0.00	0.016
*Phocaeicola abscessus*	0.18 ± 0.09	0.00 ± 0.00	0.001
*Moryella_uncultured bacterium*	0.15 ± 0.11	0.00 ± 0.00	0.002
*Family XIII_uncultured bacterium*	0.15 ± 0.05	0.01 ± 0.01	0.001
*Mycoplasma hyosynoviae*	0.12 ± 0.06	0.00 ± 0.00	0.006
*W5053_uncultured bacterium*	0.12 ± 0.07	0.00 ± 0.00	0.009
*Flexilinea_Unclassified*	0.11 ± 0.04	0.01 ± 0.00	0.003
*Bulleidia_Unclassified*	0.10 ± 0.09	0.00 ± 0.00	0.009
**Lower in AB**			
Streptococcus_uncultured bacterium	3.42 ± 1.40	13.21 ± 2.24	< 0.001
Streptococcus_Unclassified	0.56 ± 0.25	6.23 ± 1.78	< 0.001
Veillonella_uncultured bacterium	0.29 ± 0.12	4.35 ± 1.38	< 0.001
Actinomyces_uncultured bacterium	0.44 ± 0.10	3.20 ± 1.21	0.001
Neisseria_Unclassified	0.11 ± 0.06	2.98 ± 1.16	0.001
Capnocytophaga_uncultured bacterium	0.26 ± 0.11	2.94 ± 0.96	< 0.001
Prevotella 7_uncultured bacterium	0.79 ± 0.49	2.03 ± 0.60	0.005
Lautropia_uncultured bacterium	1.52 ± 1.47	1.68 ± 0.66	0.005
*Gemella_uncultured bacterium*	0.81 ± 0.58	1.63 ± 0.47	< 0.001
*Leptotrichia_uncultured bacterium*	0.12 ± 0.04	1.45 ± 0.34	< 0.001
*F0332_uncultured bacterium*	0.01 ± 0.01	1.13 ± 0.60	0.005
*Granulicatella_uncultured bacterium*	0.06 ± 0.02	0.81 ± 0.18	< 0.001
*Selenomonas 3_uncultured bacterium*	0.06 ± 0.05	0.81 ± 0.28	0.011
*Blastomonas_uncultured bacterium*	0.00 ± 0.00	0.52 ± 0.23	0.006
*Ruminococcaceae UCG-014_uncultured bacterium*	0.00 ± 0.00	0.45 ± 0.23	0.006
*Kingella_uncultured bacterium*	0.00 ± 0.00	0.44 ± 0.20	< 0.001
*Delftia_Unclassified*	0.00 ± 0.00	0.29 ± 0.13	0.011
*Flavobacterium_Unclassified*	0.04 ± 0.02	0.25 ± 0.08	0.011
*Brevundimonas_uncultured bacterium*	0.01 ± 0.01	0.24 ± 0.07	0.002
*Aggregatibacter_uncultured bacterium*	0.00 ± 0.00	0.22 ± 0.11	0.012
*Ralstonia_Unclassified*	0.00 ± 0.00	0.18 ± 0.06	0.012
*Lachnoanaerobaculum_uncultured bacterium*	0.01 ± 0.00	0.14 ± 0.05	0.009

*Abundance cutoff was set at 0.1%, some below 0.1% were not shown in the table.*

*Porphyromonas gingivalis* and *P. intermedia* were found to be dominant in the AB group at the patient level, and their abundance was significantly higher in the AB group than in the HC group at the group level. Additionally, *Prevotella* spp. was identified to be the dominant species in the abscesses of a few patients, but its abundance was not significantly different between the AB and HC groups and it could not be classified at the species level. In contrast, *Streptococcus* spp., *Actinomyces* spp., *Lautropia* spp., *Neisseria* spp., and *Veillonella* spp. were the dominant species in the PC group compared with those in the AB group at the patient level, and their abundance was significantly lower in the AB group than in the HC group at the group level.

### Specific Bacteria Associated With Periodontitis at the Group Level

We also compared the bacterial abundance between the PO and HC groups at the group level to determine if our data were consistent with well-known periodontitis-associated bacteria. At the genus level, 23 genera including genus *Porphyromonas*, *Treponema 2*, *Tannerella*, *Fretibacterium*, *Prevotella 1*, *Filifactor*, *Dialister*, and *Desulfobulbus* were significantly higher in the PO group than in the HC group, whereas 17 genera including *Streptococcus*, *Bacteroides*, *Veillonella*, *Bergeyella*, and *Kingella* were lower in the PO group than in the HC group (Supplementary Table S4). At the OTU level, the abundance of 29 OTUs including *P. gingivalis*, *P. intermedia*, *Treponema* spp., *T. forsythia*, *F. alocis*, and *P. heparinolytica* were higher in the PO group than in the HC group. The results were partly concordant with a previous study about the periodontal red and orange complex (Newman et al., 2015). In contrast, the abundance of 23 OTUs including *Streptococcus* spp. and *B. neonati* were lower in the PO group than in the HC group (Supplementary Table S5).

## Discussion

Oral periodontal abscess is an oral infective, painful disease that can spread (Yoneda et al., 2011; Herrera et al., 2014; Sato et al., 2016), and it is a valuable potential sign of undiagnosed type 2 diabetes (Alagl, 2017). Several studies have identified the dominant microbiota by culture-based diagnostic methods (Newman and Sims, 1979; Jaramillo et al., 2005; Xie et al., 2014). In the present study, considering the heterogeneity of dominant opportunistic bacteria in different patients, a patient level analysis between abscess and healthy periodontium was made, which showed that *P. gingivalis*, and *Prevotella* spp. including *P. intermedia* were found to be dominant in the abscess of some patients compared to those of healthy periodontium based on 16S rDNA metagenomic sequencing. Compared to the findings of our previous culture-based study, our study confirmed that *Prevotella* spp., and especially *P. intermedia*, is the dominant species in human periodontal abscess (Xie et al., 2014).

However, this study differs from the traditional culture-based method in the following aspects. First, the culture method usually detects the most abundant bacterium, but the second or the third most abundant bacteria can be neglected. For example, in abscess samples from two patients, *P*. *gingivalis W83* was the most dominant bacterium, and the abundance was 39.4 and 28.5%, respectively. Furthermore, the second highest abundant bacterium in both samples was *Fusobacterium* spp., for which abundance was only 12.3 and 17.9%, respectively, which could be neglected in the culture method. Second, the metagenomic sequencing method can detect unculturable bacteria and is not restricted to medium selectivity or addictive antibiotics. For example, in one abscess sample, the dominant bacterium was *Treponema 2* spp. (Table 2), which is unculturable. In addition, the major dominant bacterium identified in the present study was *P. gingivalis*, which was not detected in some by the culture method (Newman and Sims, 1979; Jaramillo et al., 2005; Xie et al., 2014). This might be attributed to medium selectivity or addition of selective antibiotics that inhibit this bacterium.

*Porphyromonas gingivalis* is a member of periodontal red complex (Socransky et al., 1998; Newman et al., 2015), which is the most predominant bacterial cluster detected in subgingival plaque, and can induce the production of interleukin-1 in macrophages (Saito et al., 1997) and trigger polyclonal B-cell activation (Champaiboon et al., 2000) associated with bleeding on probing and alveolar bone loss (Socransky et al., 1998). Moreover, it might be associated with several general dysfunctions including cardiovascular disease (Kozarov et al., 2005), rheumatoid arthritis (Berthelot and Le Goff, 2010), Alzheimer’s disease (Dominy et al., 2019), and conception in women (Paju et al., 2017). *Prevotella* spp. including *P. intermedia* is a member of the periodontal orange complex (Newman et al., 2015), the second most predominant bacterial cluster detected in subgingival plaque, in addition to being recognized pathogens of periodontal infection. In a study by Jaramillo et al. (2005), the most frequent subgingival bacterium was *Fusobacterium* spp. (75%), followed by *P. intermedia* and *P. nigrescens* (60%), as well as *P. gingivalis* (51%). In partial agreement of the results of our previous study that *P. intermedia* is the most prevalent bacterium in periodontal abscess (Xie et al., 2014), in the present study, the second and third most dominant bacteria were *Prevotella* spp. and *P. intermedia* (25%).

Like brain, lung, and pyogenic liver abscesses, which are caused by multiple kinds of bacteria (Brook, 2009; Webb et al., 2014; Yazbeck et al., 2014), periodontal abscess is more complex than previously thought. In the present study, seven of 20 samples had three or more OTUs with an abundance greater than 10%, and most OTUs were opportunistic bacteria, suggesting pathogen heterogeneity and bacterial co-infection in periodontal abscess diseases. Abscess occurs in a site that inhabits multiple normal and opportunistic bacteria, which are symbiotic and promote abscess formation (Newman et al., 2015). The significantly dominant bacteria in the abscess were also diverse in different patients and the difference between the abscess and the pocket remains unknown.

To the best of our knowledge, the bacteria in the periodontal abscess and periodontal pocket were compared for the first time. Periodontal abscess might represent acute exacerbation of periodontitis that is favored by changes in the subgingival microbiota, with an increase in bacterial virulence or a decrease in host defense (Herrera et al., 2014), resulting in the disruption of chronic phase (pocket) homeostasis and conversion to the acute phase (abscess). It is noteworthy that only two OTUs, *F. alocis* and *A. rimae*, were significantly higher in abundance in the AB group than in the PO group at the group level, indicating that they could be associated with the exacerbation of chronic periodontitis to acute periodontal abscess, although bacterial abundance and diversity were highly similar between the AB and PO groups. *F. alocis* is a Gram-positive anaerobic rod, which is now suggested to be a new periodontal pathogen (Schlafer et al., 2010; Aruni et al., 2015; Camelo-Castillo et al., 2015) with unique properties such as resistance to oxidative stress (Aruni et al., 2011), the ability to cause chronic inflammation (Fine et al., 2013), and the capacity to trigger apoptosis of gingival epithelial cells (Moffatt et al., 2011). In the present study, consistent with the findings of previous studies that *F. alocis* is positively associated with periodontitis, *F. alocis* was found to be more abundant in periodontal pockets than in the healthy periodontium. Furthermore, it was more abundant in periodontal abscess than in the pocket, suggesting that it is a potential, acute abscess-related, periodontal pathogen. *A. rimae* is an anaerobic, Gram-positive, rod-shaped bacterium, which has been suggested to be an endodontic abscess-related microorganism (Tennert et al., 2014; George et al., 2016). In the present study, *A. rimae* was found to be more significantly abundant in the abscess than in the pocket and healthy periodontium of the same patient. However, it has been reported that *A. rimae* is more prevalent in healthy subjects (Kumar et al., 2003), suggesting its role in periodontitis formation, which is complex and requires further study.

The bacteria associated with periodontitis have been well investigated previously (Liu et al., 2012; Wang et al., 2013). In the present study, the finding that the abundance of 29 OTUs including *P. gingivalis*, *P. intermedia*, *T. forsythia*, and *F. alocis* was higher in the PO group than in the HC group was largely in agreement with the findings of previous studies (Liu et al., 2012; Wang et al., 2013). These data further strengthen the reliability of this study to investigate the opportunistic pathogens and dominant microbiota associated with periodontal abscess.

Meanwhile, there were some limitations to our study. First, only the V4–V5 region, and not the full-length gene, was sequenced, which might result in some unclassified OTUs like *Streptococcus_Unclassified* at the species level. Second, different bacterial databases could lead to differences in detected species, which requires further comparisons with previously published studies to confirm the suitability of the present research. Third, the present study did not quantify the bacterial loads in samples, and quantitative research will be more helpful in unraveling the relationship between the severity of periodontal abscess and certain bacteria.

In conclusion, we used 16S rRNA-based metagenomics to characterize the bacterial profile of periodontal abscess in humans and compared it with the corresponding periodontal pocket and healthy periodontium. The results showed that the bacterial composition of periodontal abscess is more complex and mainly involves bacterial co-infections. Further, *P. gingivalis*, *P. intermedia*, and *Prevotella* spp. were the predominant bacteria in human periodontal abscesses. Two species, *F. alocis* and *A. rimae*, were found to be positively associated with abscess formation, although their bacterial abundance and diversity in periodontal abscess and periodontal pockets were highly similar. Recognition of the bacterial profile of periodontal abscess might reveal new strategies for the diagnosis, surveillance, and treatment of periodontal abscess, including the accurate use of antibiotics and probiotics.

## Data Availability

All sequencing data were uploaded and deposited to the SRA database with project number PRJNA547446.

## Ethics Statement

This study was approved by the Ethics Committee of the Huashan Hospital, Fudan University (No. KY2014-023).

## Author Contributions

JC and XW conceived and designed the study, acquired, analyzed, and interpreted the data, and drafted and critically revised the manuscript. DZ, MX, and YY acquired the data and critically revised the manuscript. LY and WZ conceived and designed the study, analyzed and interpreted the data, and drafted and critically revised the manuscript. All authors approved the final manuscript and agreed to be accountable for all aspects of the work.

## Conflict of Interest Statement

The authors declare that the research was conducted in the absence of any commercial or financial relationships that could be construed as a potential conflict of interest.

## Footnotes

1 http://rdp.cme.msu.edu/
