# Supplementary material for: Microbiota in Human Periodontal Abscess Revealed by 16S rDNA Sequencing
Source: Front Microbiol. 2019 Jul 30;10:1723. doi: 10.3389/fmicb.2019.01723 (PMC6682650; doi:10.3389/fmicb.2019.01723)
Supplement: Supplementary file 1 [file Data_Sheet_1.docx]

Participants (N=45)

Health control (N=25)

Abscess patients (N=20)

Isolation of bacterial DNA

Sample collection

Amplification of the 16S rDNA gene by PCR

Illumina sequencing

Microbiome analysis

Abundance analysis

Beta-diversity analysis

Alpha-diversity analysis

Taxonomic classification

**Figure S1. Flowchart of the study design.**

A total of 45 participants were recruited, including 20 patients with periodontal abscess and 25 health controls. After samples collection, isolation of bacterial DNA, preparation for PCR and 16s sequencing, sequence data was analyzed with taxonomic classification, alpha-diversity analysis, beta-diversity analysis and abundance analysis.

**
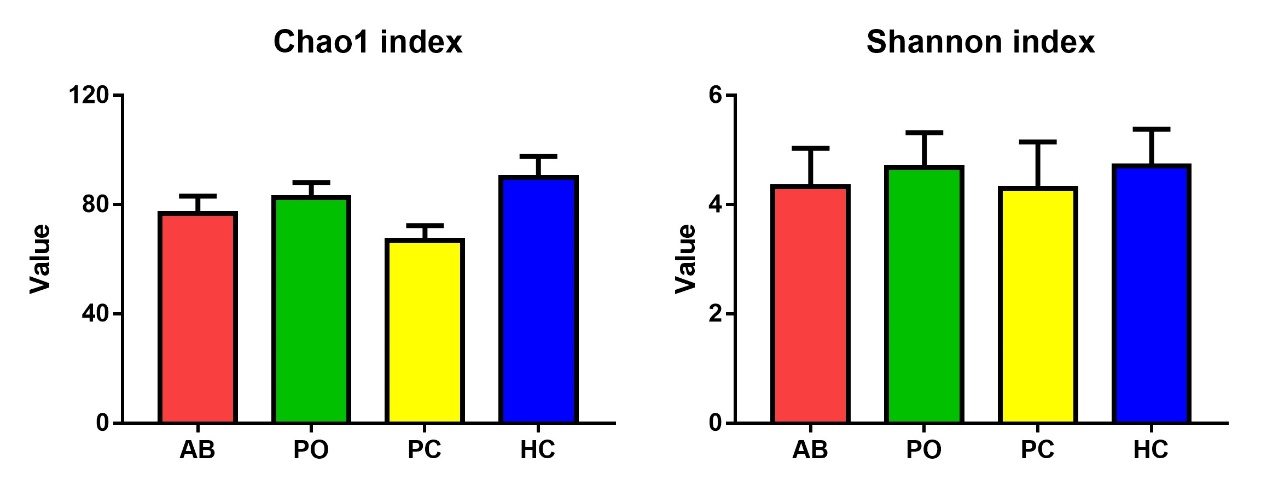
**

**Figure S2. Chao1 and Shannon index of four groups.** The data was shown in mean (SE). Significant difference was based on the one-way ANOVA analysis with Tukey's multiple comparisons test. There was no significant difference of Chao1 and Shannon indexes among four groups.

**
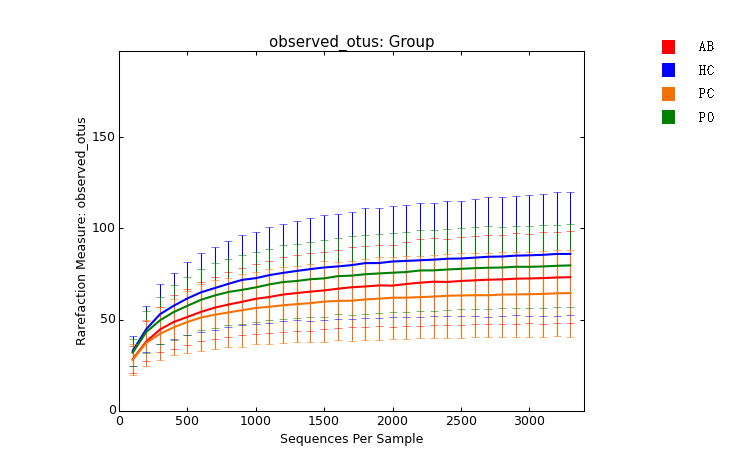
**

**Figure S3. Alpha diversity analysis based on subsampling analysis from four groups.**

Red lines indicate abscess group, blue lines indicate health control group, yellow lines indicate patient control group and green lines indicate pocket group. Representative rarefaction curves depicting the richness (y-axis, as the number of OTUs) determined at an equal sampling effort (x-axis, the number of reads recovered in samples) of periodontal microbes. OTU reads of samples from each group were standardized.

**
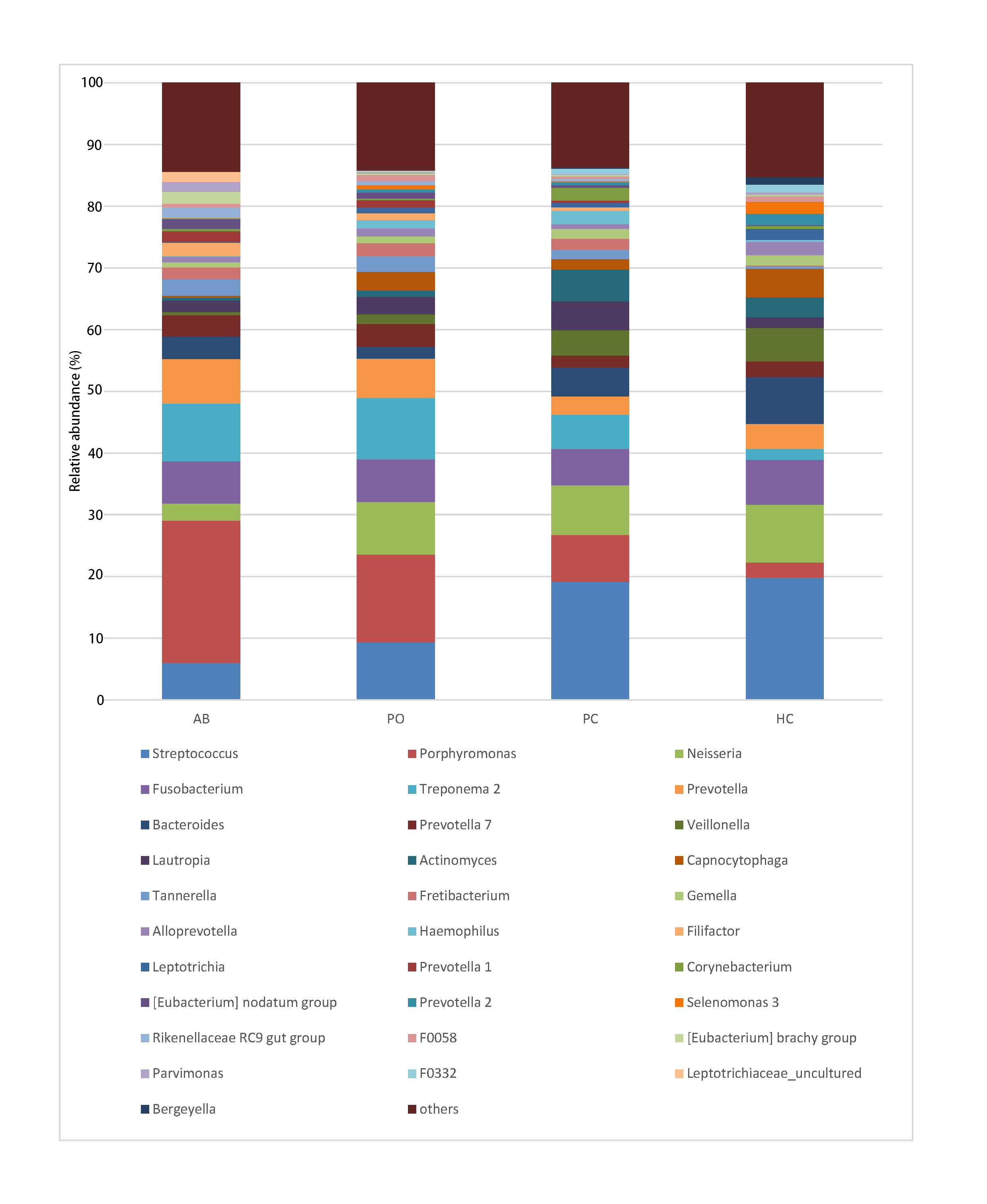
**

**Figure S4.** **Relative abundance of microbial composition at genus level.** All sequences were submitted to RDP Classifier for taxonomic identification. Abundance cutoff in this figure was set at 1%, some below 1% were shown as “others”. Each column represents a group and the abundance of detected genus was averaged in each group.

**Table S1. Specific OTUs of significant difference in AB compared with PC group based on a paired *t*-test at group level.**

| OTUs | AB (Mean±SE, %) | | | PC (Mean±SE, %) | | | p-value |
| --- | --- | --- | --- | --- | --- | --- | --- |
| **Higher in AB** |  |  |  |  |  |  |  |
| *Porphyromonas gingivalis* *W83* | 12.08 | ± | 3.07 | 7.00 | ± | 1.84 | 0.013 |
| *Filifactor alocis ATCC 35896* | 1.52 | ± | 0.40 | 0.86 | ± | 0.23 | 0.037 |
| *Pyramidobacter piscolens* | 1.43 | ± | 0.43 | 0.00 | ± | 0.00 | 0.039 |
| *Atopobium rimae* | 1.00 | ± | 0.30 | 0.02 | ± | 0.01 | 0.044 |
| *Dialister_uncultured organism* | 0.79 | ± | 0.24 | 0.09 | ± | 0.03 | 0.047 |
| *[Eubacterium] nodatum* | 0.77 | ± | 0.21 | 0.53 | ± | 0.18 | 0.020 |
| **Lower in AB** |  |  |  |  |  |  |  |
| *Streptococcus_uncultured bacterium* | 4.27 | ± | 1.43 | 12.08 | ± | 3.80 | 0.040 |
| *Actinomyces_uncultured bacterium* | 0.35 | ± | 0.10 | 6.57 | ± | 2.08 | 0.035 |
| *Veillonella_uncultured bacterium* | 0.41 | ± | 0.13 | 3.74 | ± | 1.13 | 0.012 |
| *Streptococcus_Unclassified* | 0.73 | ± | 0.26 | 2.85 | ± | 0.91 | 0.024 |
| *Granulicatella_uncultured bacterium* | 0.07 | ± | 0.02 | 0.52 | ± | 0.13 | 0.007 |
| *Campylobacter_uncultured bacterium* | 0.06 | ± | 0.02 | 0.27 | ± | 0.08 | 0.035 |

Abundance cutoff was set at 0.1%, some below 0.1% were not shown in the table.

**Table S2. The Dominant genera in AB group compared to PC group at patient level.**

| Genera | Patients（Radio） |
| --- | --- |
| **Greater than 10% higher in AB group than in PC group** |  |
| *Porphyromonas* | 11 (55%) |
| *Prevotella* | 5 (25%) |
| *Treponema 2* | 5 (25%) |
| *Fusobacterium* | 2 (10%) |
| *Streptococcus* | 2 (10%) |
| *Prevotella 1* | 1 (5.0%) |
| *Prevotella 7* | 1 (5.0%) |
| *Bacteroides* | 1 (5.0%) |
| *[Eubacterium] brachy group* | 1 (5.0%) |
| *Parvimonas* | 1 (5.0%) |
| *Leptotrichiaceae_uncultured* | 1 (5.0%) |
| *Rikenellaceae RC9 gut group* | 1 (5.0%) |
| *Tannerella* | 1 (5.0%) |
| **Greater than 10% lower in AB group than in PC group** |  |
| *Streptococcus* | 8 (40%) |
| *Treponema 2* | 3 (15%) |
| *Actinomyces* | 3 (15%) |
| *Lautropia* | 3 (15%) |
| *Neisseria* | 3 (15%) |
| *Veillonella* | 3 (15%) |
| *Prevotella* | 2 (10%) |
| *Fusobacterium* | 2 (10%) |
| *Bacteroides* | 2 (10%) |
| *Porphyromonas* | 1 (5.0%) |
| *Bergeriella* | 1 (5.0%) |
| *Capnocytophaga* | 1 (5.0%) |
| *Corynebacterium* | 1 (5.0%) |
| *F0332* | 1 (5.0%) |
| *Fretibacterium* | 1 (5.0%) |
| *Gemella* | 1 (5.0%) |
| *Haemophilus* | 1 (5.0%) |
| *Lentimicrobiaceae_norank* | 1 (5.0%) |

The dominant different abundance of genus was set as 10%.

**Table S3. Mean relative abundance of genera with significant statistical difference between AB and HC groups at group level.**

| Genera | AB (Mean±SE, %) | | | HC (Mean±SE, %) | | | p-value |
| --- | --- | --- | --- | --- | --- | --- | --- |
| **Higher in AB** |  |  |  |  |  |  |  |
| *Porphyromonas* | 22.99 | ± | 3.82 | 2.45 | ± | 0.76 | <0.001 |
| *Treponema 2* | 9.36 | ± | 2.04 | 1.71 | ± | 0.66 | <0.001 |
| *Tannerella* | 2.61 | ± | 0.72 | 0.31 | ± | 0.12 | <0.001 |
| *Filifactor* | 2.08 | ± | 0.38 | 0.09 | ± | 0.03 | <0.001 |
| *Fretibacterium* | 1.96 | ± | 0.64 | 0.24 | ± | 0.09 | 0.002 |
| *[Eubacterium] brachy group* | 1.96 | ± | 1.32 | 0.15 | ± | 0.07 | 0.010 |
| *Lautropia* | 1.85 | ± | 1.47 | 1.76 | ± | 0.66 | 0.008 |
| *Rikenellaceae RC9 gut group* | 1.77 | ± | 1.05 | 0.00 | ± | 0.00 | <0.001 |
| *Prevotella 1* | 1.77 | ± | 0.87 | 0.01 | ± | 0.01 | 0.003 |
| *Parvimonas* | 1.60 | ± | 0.73 | 0.45 | ± | 0.17 | 0.033 |
| *[Eubacterium] nodatum group* | 1.55 | ± | 0.37 | 0.10 | ± | 0.04 | <0.001 |
| *Mogibacterium* | 0.81 | ± | 0.27 | 0.18 | ± | 0.08 | 0.027 |
| *[Eubacterium] saphenum group* | 0.59 | ± | 0.27 | 0.02 | ± | 0.01 | 0.002 |
| *Defluviitaleaceae UCG-011* | 0.29 | ± | 0.14 | 0.03 | ± | 0.02 | 0.002 |
| *Clostridiales vadinBB60 group_norank* | 0.22 | ± | 0.08 | 0.01 | ± | 0.01 | 0.003 |
| *Family XIII_uncultured* | 0.19 | ± | 0.09 | 0.01 | ± | 0.01 | 0.001 |
| *Pseudoramibacter* | 0.19 | ± | 0.09 | 0.00 | ± | 0.00 | 0.016 |
| *Phocaeicola* | 0.18 | ± | 0.09 | 0.00 | ± | 0.00 | 0.001 |
| *Mycoplasma* | 0.16 | ± | 0.07 | 0.00 | ± | 0.00 | <0.001 |
| *Moryella* | 0.15 | ± | 0.11 | 0.00 | ± | 0.00 | 0.002 |
| *W5053* | 0.12 | ± | 0.07 | 0.00 | ± | 0.00 | 0.006 |
| *Flexilinea* | 0.11 | ± | 0.04 | 0.01 | ± | 0.00 | 0.003 |
| *Bulleidia* | 0.10 | ± | 0.09 | 0.00 | ± | 0.00 | 0.009 |
| **Lower in AB** |  |  |  |  |  |  |  |
| *Streptococcus* | 6.01 | ± | 1.73 | 19.81 | ± | 2.80 | <0.001 |
| *Neisseria* | 2.80 | ± | 1.98 | 9.38 | ± | 2.68 | 0.001 |
| *Veillonella* | 0.49 | ± | 0.16 | 5.39 | ± | 1.54 | <0.001 |
| *Capnocytophaga* | 0.33 | ± | 0.12 | 4.61 | ± | 1.76 | <0.001 |
| *Actinomyces* | 0.50 | ± | 0.11 | 3.27 | ± | 1.22 | 0.002 |
| *Selenomonas 3* | 0.13 | ± | 0.06 | 1.94 | ± | 0.97 | 0.005 |
| *Prevotella 2* | 0.08 | ± | 0.03 | 1.93 | ± | 0.74 | 0.015 |
| *Leptotrichia* | 0.15 | ± | 0.05 | 1.75 | ± | 0.35 | <0.001 |
| *Gemella* | 0.81 | ± | 0.58 | 1.63 | ± | 0.47 | <0.001 |
| *F0332* | 0.03 | ± | 0.02 | 1.22 | ± | 0.65 | 0.032 |
| *Bergeyella* | 0.05 | ± | 0.02 | 1.21 | ± | 0.92 | 0.003 |
| *Kingella* | 0.03 | ± | 0.03 | 0.87 | ± | 0.38 | <0.001 |
| *Granulicatella* | 0.27 | ± | 0.17 | 0.85 | ± | 0.18 | <0.001 |
| *Elizabethkingia* | 0.01 | ± | 0.01 | 0.81 | ± | 0.33 | <0.001 |
| *Blastomonas* | 0.00 | ± | 0.00 | 0.72 | ± | 0.25 | 0.001 |
| *Chryseobacterium* | 0.04 | ± | 0.02 | 0.49 | ± | 0.15 | 0.028 |
| *Ruminococcaceae UCG-014* | 0.00 | ± | 0.00 | 0.45 | ± | 0.23 | 0.006 |
| *Brevundimonas* | 0.12 | ± | 0.07 | 0.45 | ± | 0.17 | 0.010 |
| *Ralstonia* | 0.01 | ± | 0.01 | 0.34 | ± | 0.16 | 0.010 |
| *Delftia* | 0.00 | ± | 0.00 | 0.29 | ± | 0.13 | 0.011 |
| *Flavobacterium* | 0.04 | ± | 0.02 | 0.27 | ± | 0.09 | 0.024 |
| *Lachnoanaerobaculum* | 0.02 | ± | 0.01 | 0.18 | ± | 0.05 | 0.006 |
| *Pedobacter* | 0.02 | ± | 0.02 | 0.15 | ± | 0.06 | 0.013 |
| *Cutibacterium* | 0.02 | ± | 0.01 | 0.12 | ± | 0.05 | 0.021 |
| *Stomatobaculum* | 0.00 | ± | 0.00 | 0.10 | ± | 0.06 | 0.012 |

Abundance cutoff was set at 0.1%, some below 0.1% were not shown in the table.

**Table S4. Mean relative abundance of genera with significant statistical difference between PO and HC groups at group level.**

| Genera | PO (Mean±SE, %) | | | HC (Mean±SE, %) | | | p-value |
| --- | --- | --- | --- | --- | --- | --- | --- |
| **Higher in PO** |  |  |  |  |  |  |  |
| *Porphyromonas* | 14.26 | ± | 2.80 | 2.45 | ± | 0.76 | <0.001 |
| *Treponema 2* | 9.95 | ± | 1.92 | 1.71 | ± | 0.66 | 0.002 |
| *Tannerella* | 2.51 | ± | 0.61 | 0.31 | ± | 0.12 | <0.001 |
| *Fretibacterium* | 2.13 | ± | 0.63 | 0.24 | ± | 0.09 | 0.010 |
| *Prevotella 1* | 1.16 | ± | 0.51 | 0.01 | ± | 0.01 | 0.013 |
| *Filifactor* | 1.06 | ± | 0.25 | 0.09 | ± | 0.03 | <0.001 |
| *[Eubacterium] nodatum group* | 0.94 | ± | 0.22 | 0.10 | ± | 0.04 | <0.001 |
| *Lentimicrobiaceae_norank* | 0.84 | ± | 0.44 | 0.51 | ± | 0.48 | 0.045 |
| *Dialister* | 0.72 | ± | 0.25 | 0.25 | ± | 0.10 | 0.038 |
| *Rikenellaceae RC9 gut group* | 0.64 | ± | 0.20 | 0.00 | ± | 0.00 | <0.001 |
| *Desulfobulbus* | 0.61 | ± | 0.22 | 0.03 | ± | 0.02 | <0.001 |
| *Flexilinea* | 0.44 | ± | 0.17 | 0.01 | ± | 0.00 | <0.001 |
| *Peptostreptococcus* | 0.43 | ± | 0.17 | 0.16 | ± | 0.07 | 0.048 |
| *Mogibacterium* | 0.40 | ± | 0.12 | 0.18 | ± | 0.08 | 0.022 |
| *[Eubacterium] saphenum group* | 0.32 | ± | 0.22 | 0.02 | ± | 0.01 | 0.039 |
| *Selenomonas 4* | 0.26 | ± | 0.10 | 0.21 | ± | 0.12 | 0.019 |
| *Defluviitaleaceae UCG-011* | 0.23 | ± | 0.09 | 0.03 | ± | 0.02 | 0.002 |
| *Phocaeicola* | 0.21 | ± | 0.10 | 0.00 | ± | 0.00 | <0.001 |
| *W5053* | 0.18 | ± | 0.10 | 0.00 | ± | 0.00 | 0.029 |
| *Family XIII_uncultured* | 0.15 | ± | 0.07 | 0.01 | ± | 0.01 | 0.002 |
| *Moryella* | 0.11 | ± | 0.06 | 0.00 | ± | 0.00 | 0.001 |
| *Butyrivibrio 2* | 0.11 | ± | 0.05 | 0.02 | ± | 0.02 | 0.024 |
| *Pseudoramibacter* | 0.11 | ± | 0.05 | 0.00 | ± | 0.00 | 0.033 |
| **Lower in PO** |  |  |  |  |  |  |  |
| *Streptococcus* | 9.32 | ± | 2.95 | 19.81 | ± | 2.80 | 0.002 |
| *Bacteroides* | 1.91 | ± | 0.93 | 7.61 | ± | 2.42 | 0.037 |
| *Veillonella* | 1.59 | ± | 0.67 | 5.39 | ± | 1.54 | 0.003 |
| *Bergeyella* | 0.11 | ± | 0.04 | 1.21 | ± | 0.92 | 0.028 |
| *Kingella* | 0.61 | ± | 0.36 | 0.87 | ± | 0.38 | 0.013 |
| *Granulicatella* | 0.70 | ± | 0.36 | 0.85 | ± | 0.18 | 0.002 |
| *Elizabethkingia* | 0.00 | ± | 0.00 | 0.81 | ± | 0.33 | <0.001 |
| *Blastomonas* | 0.00 | ± | 0.00 | 0.72 | ± | 0.25 | <0.001 |
| *Chryseobacterium* | 0.00 | ± | 0.00 | 0.49 | ± | 0.15 | 0.001 |
| *Brevundimonas* | 0.03 | ± | 0.02 | 0.45 | ± | 0.17 | 0.001 |
| *Ralstonia* | 0.00 | ± | 0.00 | 0.34 | ± | 0.16 | 0.001 |
| *Delftia* | 0.01 | ± | 0.01 | 0.29 | ± | 0.13 | 0.017 |
| *Flavobacterium* | 0.02 | ± | 0.01 | 0.27 | ± | 0.09 | 0.002 |
| *Pedobacter* | 0.01 | ± | 0.01 | 0.15 | ± | 0.06 | 0.017 |
| *Cutibacterium* | 0.01 | ± | 0.01 | 0.12 | ± | 0.05 | 0.003 |
| *Stenotrophomonas* | 0.06 | ± | 0.06 | 0.11 | ± | 0.03 | 0.009 |
| *Achromobacter* | 0.00 | ± | 0.00 | 0.11 | ± | 0.04 | 0.001 |

Abundance cutoff was set at 0.1%, some below 0.1% were not shown in the table.

**Table S5. Mean relative abundance of OTUs with significant statistical difference between PO and HC groups at group level.**

| OTUs | PO (Mean±SE, %) | | | HC (Mean±SE, %) | | | p-value |
| --- | --- | --- | --- | --- | --- | --- | --- |
| **Higher in PO** |  |  |  |  |  |  |  |
| *Porphyromonas gingivalis W83* | 7.58 | ± | 2.41 | 0.09 | ± | 0.04 | 0.001 |
| *Treponema 2_Unclassified* | 4.60 | ± | 1.10 | 0.75 | ± | 0.30 | 0.005 |
| *Treponema 2_uncultured Treponema sp.* | 3.02 | ± | 0.82 | 0.08 | ± | 0.03 | 0.000 |
| *Prevotella intermedia* | 2.70 | ± | 0.89 | 0.17 | ± | 0.07 | 0.005 |
| *Fretibacterium_Unclassified* | 2.05 | ± | 0.63 | 0.20 | ± | 0.09 | 0.008 |
| *Tannerella forsythia KS16* | 1.96 | ± | 0.62 | 0.05 | ± | 0.03 | 0.002 |
| *Prevotella 7_Unclassified* | 1.95 | ± | 0.81 | 0.39 | ± | 0.16 | 0.003 |
| *Treponema 2_uncultured bacterium* | 1.83 | ± | 0.51 | 0.46 | ± | 0.19 | 0.003 |
| *Porphyromonas_uncultured organism* | 1.73 | ± | 0.58 | 0.00 | ± | 0.00 | 0.000 |
| *Prevotella 1_Unclassified* | 1.16 | ± | 0.51 | 0.01 | ± | 0.01 | 0.013 |
| *[Eubacterium] nodatum* | 0.89 | ± | 0.22 | 0.03 | ± | 0.02 | 0.000 |
| *Lentimicrobiaceae_uncultured bacterium* | 0.80 | ± | 0.44 | 0.51 | ± | 0.48 | 0.048 |
| *Filifactor alocis ATCC 35896* | 0.77 | ± | 0.23 | 0.05 | ± | 0.03 | 0.001 |
| *Desulfobulbus_Unclassified* | 0.61 | ± | 0.22 | 0.03 | ± | 0.02 | 0.000 |
| *Flexilinea_Unclassified* | 0.44 | ± | 0.17 | 0.01 | ± | 0.00 | 0.000 |
| *Rikenellaceae RC9 gut group_Unclassified* | 0.43 | ± | 0.15 | 0.00 | ± | 0.00 | 0.000 |
| *Peptostreptococcus_uncultured organism* | 0.43 | ± | 0.17 | 0.16 | ± | 0.07 | 0.048 |
| *Eubacterium saphenum ATCC 49989* | 0.32 | ± | 0.22 | 0.02 | ± | 0.01 | 0.039 |
| *Defluviitaleaceae UCG-011_Unclassified* | 0.22 | ± | 0.09 | 0.03 | ± | 0.02 | 0.004 |
| *Prevotella heparinolytica* | 0.21 | ± | 0.14 | 0.00 | ± | 0.00 | 0.045 |
| *Phocaeicola abscessus* | 0.21 | ± | 0.10 | 0.00 | ± | 0.00 | 0.000 |
| *Campylobacter_uncultured bacterium* | 0.18 | ± | 0.09 | 0.01 | ± | 0.01 | 0.042 |
| *Rikenellaceae RC9 gut group_uncultured bacterium* | 0.17 | ± | 0.10 | 0.00 | ± | 0.00 | 0.005 |
| *Parvimonas_uncultured bacterium* | 0.17 | ± | 0.06 | 0.02 | ± | 0.01 | 0.000 |
| *W5053_uncultured bacterium* | 0.16 | ± | 0.10 | 0.00 | ± | 0.00 | 0.045 |
| *Peptococcus_Unclassified* | 0.15 | ± | 0.07 | 0.01 | ± | 0.01 | 0.033 |
| *Moryella_uncultured bacterium* | 0.11 | ± | 0.06 | 0.00 | ± | 0.00 | 0.001 |
| *Butyrivibrio 2_Unclassified* | 0.11 | ± | 0.05 | 0.02 | ± | 0.02 | 0.024 |
| *Family XIII_uncultured bacterium* | 0.10 | ± | 0.03 | 0.01 | ± | 0.01 | 0.006 |
| **Lower in PO** |  |  |  |  |  |  |  |
| *Streptococcus_uncultured bacterium* | 7.57 | ± | 2.70 | 13.21 | ± | 2.24 | 0.008 |
| *Bacteroides neonati* | 1.56 | ± | 0.91 | 7.29 | ± | 2.34 | 0.001 |
| *Streptococcus_Unclassified* | 1.30 | ± | 0.50 | 6.23 | ± | 1.78 | 0.002 |
| *Veillonella_uncultured bacterium* | 1.42 | ± | 0.61 | 4.35 | ± | 1.38 | 0.036 |
| *Actinomyces_uncultured bacterium* | 0.89 | ± | 0.23 | 3.20 | ± | 1.21 | 0.038 |
| *Neisseria_Unclassified* | 2.19 | ± | 1.76 | 2.98 | ± | 1.16 | 0.014 |
| *Prevotella 7_uncultured bacterium* | 1.19 | ± | 0.85 | 2.03 | ± | 0.60 | 0.016 |
| *Leptotrichia_uncultured bacterium* | 0.55 | ± | 0.20 | 1.45 | ± | 0.34 | 0.024 |
| *Veillonella_uncultured organism* | 0.15 | ± | 0.09 | 0.86 | ± | 0.26 | 0.043 |
| *Granulicatella_uncultured bacterium* | 0.69 | ± | 0.37 | 0.81 | ± | 0.18 | 0.011 |
| *Blastomonas_uncultured bacterium* | 0.00 | ± | 0.00 | 0.52 | ± | 0.23 | 0.008 |
| *Elizabethkingia_Unclassified* | 0.00 | ± | 0.00 | 0.50 | ± | 0.28 | 0.014 |
| *Kingella_Unclassified* | 0.00 | ± | 0.00 | 0.43 | ± | 0.23 | 0.025 |
| *Parvimonas_uncultured organism* | 0.03 | ± | 0.02 | 0.41 | ± | 0.17 | 0.027 |
| *Delftia_Unclassified* | 0.01 | ± | 0.01 | 0.29 | ± | 0.13 | 0.017 |
| *Chryseobacterium_Unclassified* | 0.00 | ± | 0.00 | 0.26 | ± | 0.08 | 0.001 |
| *Elizabethkingia meningoseptica* | 0.00 | ± | 0.00 | 0.25 | ± | 0.20 | 0.025 |
| *Flavobacterium_Unclassified* | 0.02 | ± | 0.01 | 0.25 | ± | 0.08 | 0.002 |
| *Brevundimonas_uncultured bacterium* | 0.00 | ± | 0.00 | 0.24 | ± | 0.07 | 0.001 |
| *Leptotrichia_Unclassified* | 0.12 | ± | 0.12 | 0.19 | ± | 0.07 | 0.039 |
| *Ralstonia_Unclassified* | 0.00 | ± | 0.00 | 0.18 | ± | 0.06 | 0.004 |
| *Pedobacter_Unclassified* | 0.01 | ± | 0.01 | 0.15 | ± | 0.06 | 0.031 |
| *Cutibacterium_uncultured bacterium* | 0.01 | ± | 0.01 | 0.12 | ± | 0.05 | 0.003 |

Abundance cutoff was set at 0.1%, some below 0.1% were not shown in the table.
